# A corpus-based analysis on the use of MAKE in sinologist Cyril Birch’s English version of *Mistress and Maid (Jiaohongji)*

**DOI:** 10.1371/journal.pone.0338015

**Published:** 2026-01-08

**Authors:** Chunli Yu, Yurong Wu

**Affiliations:** 1 School of Foreign Languages, Qinghai Normal University, Xining, Qinghai, China; 2 Faculty of Forestry and Environment, Universiti Putra Malaysia, Selangor, Malaysia; Southwest University, CHINA

## Abstract

While corpus-based translation studies have extensively analyzed lexical patterns, they exhibit critical theoretical limitations in integrating translation theories with empirical analysis, particularly in dramatic translation contexts where cultural mediation and audience reception intersect. Existing high-frequency verb research lacks systematic analysis of how individual lexical choices serve multiple simultaneous function—linguistic, cultural, and theatrical—in cross-cultural dramatic translation. This study addresses these gaps through integrated corpus-translation theory analysis of MAKE deployment in Cyril Birch’s English translation of *Mistress and Maid (Jiaohongji)*, a classical Chinese chuanqi play. Using AntConc 4.1.1 for corpus analysis (158 MAKE instances from 86,047-word corpus) combined with systematic cultural context examination, the research investigates how high-frequency verbs function as cultural bridging mechanisms. Methodological limitations include single-translator focus and potential genre-specific constraints. Analysis reveals five systematic functional categories: delexical verb constructions facilitating cultural concept transfer, causative verb patterns in bridging metaphorical traditions, notional verb usage in supporting plot development, linking constructions creating thematic resonance, and phrasal verb constructions in emotional expression. These patterns demonstrate that high-frequency verb deployment serves strategic cultural mediation functions beyond simple linguistic substitution. Theoretical Contributions: the study validates and refines the application translation universals in dramatic contexts, demonstrating how simplification and explicitation operate through systematic high-frequency verb deployment. It integrates descriptive translation studies with cultural mediation theory, revealing how micro-level lexical choices serve macro-level intercultural communication goals while challenging corpus linguistics’ purely quantitative approaches to translation analysis. Findings offer evidence-based guidance for dramatic translators regarding strategic use of high-frequency verbs to balance source-text fidelity with target-audience accessibility, particularly in contexts requiring cultural sensitivity and dramatic authenticity.

## 1. Introduction

The fundamental role of verbs in linguistic structure and meaning-making has long been recognized in both theoretical linguistics and translation studies. Within the vast category of verbs, a small subset of high-frequency verbs demonstrates remarkable prevalence and complexity across languages. Altenberg and Granger’s (2001) seminal research identifies fifteen core high-frequency verbs in English, including “make”, which maintain consistent presence across diverse corpora [1]. These verbs, as Viberg (1996) demonstrates, exhibit distinctive characteristics: they dominate various semantic fields, maintain cross-linguistic equivalence, display complex polysemous patterns, and present significant challenges in second language acquisition and translation practice [2].

While extensive research has examined high-frequency verbs in the context of language learning, their role in translated texts, particularly in dramatic translation, remains understudied. This research gap becomes particularly significant when considered alongside Baker’s (1996) concept of “translation universals”, distinctive linguistic patterns that characterize translated texts [3]. The deployment of high-frequency verbs in translation not only reveals translators’ linguistic preferences but also illuminates their strategic approaches to cultural and dramatic adaptation. This perspective becomes especially relevant in the context of theatrical translation, where linguistic choices must serve both semantic and dramatic functions.

Dramatic texts present unique challenges that differentiate them from other forms of literary translation. The intricate interplay between dialogue and stage directions creates a complex linguistic environment where high-frequency verbs must simultaneously convey meaning, maintain dramatic tension, and preserve cultural authenticity. This complexity is further amplified in cross-cultural translations, such as rendering Chinese classical play into English, where cultural elements, theatrical conventions, and linguistic patterns differ significantly.

The present study examines the deployment of MAKE (including its inflectional forms “makes”, “made”, and “making”) in Cyril Birch’s (2001) English translation of *Mistress and Maid (Jiaohongji)* [4]. Through corpus-based analysis, this research investigates both quantitative patterns of usage and qualitative aspects of functional deployment. The study addresses two primary research questions: First, what patterns emerge in the frequency and distribution of MAKE in this dramatic translation? Second, how do different functional categories of MAKE serve both linguistic and theatrical purposes in the translated text?

This investigation contributes to both translation studies and linguistic research by examining how high-frequency verbs function as linguistic bridges in cross-cultural dramatic translation. The study employs corpus linguistics tools to provide empirical evidence for usage patterns while considering the broader context of dramatic and cultural translation. The findings have implications for translation practice, particularly in contexts requiring balanced attention to linguistic precision and dramatic effectiveness. Through this analysis, the study aims to enhance our understanding of how high-frequency verbs contribute to successful dramatic translation, particularly in works that bridge significantly different theatrical traditions. This research not only addresses a gap in current scholarship but also provides practical insights for translators working with classical theatrical texts.

## 2. Literature review

Research on high frequency verbs has drawn considerable attention in linguistics and translation studies. The terminology used to describe these words has evolved over time, reflecting different theoretical perspectives and research focuses. Jespersen (1942) introduced the term “light verb” to describe verbs with reduced semantic content that primarily serve grammatical functions [5]. Later studies have employed various terms including “delexical words” and “high frequency verbs” to capture different aspects of these linguistic phenomena [1,6].

The concept of translation universals, as proposed by Baker (1996), provides a theoretical framework for understanding how these high frequency verbs behave in translated texts. Baker argues that certain features are inherent in translated texts regardless of the language pairs involved, including simplification, explicitation, and normalization. These universal features may influence translators’ choices in handling high frequency verbs like MAKE [3].

Delexical words, as Gui (2007) points out, can belong to various parts of speech including nouns, verbs, adjectives, adverbs, and prepositions. Of particular relevance to this study is observation that delexical verbs typically overlap with high frequency verbs [7]. Contemporary scholars have identified a core set of six high frequency verbs in English: “do, make, have, take, give, get” [8]. These verbs demonstrate remarkable versatility in their semantic and syntactic functions, often presenting significant challenges in translation.

The present study adopts the term “high frequency verbs” rather than “delexical words” for two primary reasons. First, it provides a more precise specification of the part of speech under investigation. Second, it better captures the full range of both semantic and grammatical functions that characterize the usage of MAKE in translation contexts. This terminological choice aligns with recent research trends in corpus-based translation studies and facilitates more systematic analysis of translation patterns [9,10].

### 2.1. High frequency verbs in EFL

The investigation of high frequency verbs has been a significant focus in English as a Foreign Language (EFL) research, particularly through corpus-based approaches. Recent studies have extensively examined learners’ usage patterns, collocational competence, and error types associated with these verbs [1,11,12].

Corpus-based analyses have revealed systematic patterns in EFL learners’ acquisition and deployment of high frequency verbs. Liang (2018) conducted a comprehensive investigation of collocational patterns with delexical verbs “do”, “make”, and “take” among senior high school English learners, identifying both successful acquisition patterns and persistent challenges [11]. Similarly, Pérez and Taouis (2019) focused specifically on noun collocations with the verb “do” in Spanish university students’ written production, demonstrating how learner corpora can illuminate cross-linguistic influence in collocation acquisition [12].

More recent research has expanded this focus to include detailed error analysis. Li (2022) employed corpus data to explore systematic errors in learners’ usage of the high frequency verb “take” [13], while Suleiman (2022) investigated Saudi EFL learners’ collocation accuracy with delexical verbs “do”, “have”, “make”, and “take”, providing valuable insights into the cognitive and linguistic factors underlying collocation errors [14]. Zhang & Zhang (2023) applied the Language Exposure Hypothesis to the Chinese EFL Beginners’ Spoken Corpus, examining collocational errors with high-frequency verbs (like, go, make) across three proficiency stages [10]. These studies collectively demonstrate that mastery of high frequency verbs remains a significant challenge for EFL learners across different proficiency levels and linguistic backgrounds.

The prevalence of corpus-based methodologies in these studies not only validates the reliability of this research approach but also enables more nuanced understanding of learner language development. This methodological consistency has facilitated comparative analyses across different learning contexts and proficiency levels, contributing to both theoretical understanding and practical pedagogical applications. The sustained scholarly attention to this area reflects its continuing significance in second language acquisition research and its practical implications for EFL pedagogy.

### 2.2. High frequency verbs in translated texts

While translation studies have increasingly recognized translated texts as a distinct linguistic variety (Baker 1996), research specifically examining high frequency verbs in translated texts remains relatively limited [3]. This research gap is particularly noteworthy given that translation, as a form of linguistic rewriting, offers unique insights into how these fundamental lexical items function across languages and cultures. Recent corpus-based studies have begun to address this gap, though often with different focuses. Recent corpus-based studies have begun addressing this gap through varied approaches. Xu and Zi (2014) conducted analysis of lexical features in translated business news, though their genre-specific focus limits applicability to literary translation [15]. Li (2015) examined delexical verb translation patterns in Shakespeare, yet this study’s unidirectional focus (English-to-Chinese) overlooks the reverse translation challenges this research examines [16]. More critically, these studies lack integration with contemporary translation theory [17,18] and fail to address dramatic translation’s unique requirements [19]. Valencia Giraldo and Corpas Pastor (2019) examined verb+noun collocations across Spanish translations, though their study was not specifically focused on high frequency verbs [20]. Their findings, however, suggest that collocational patterns in translated texts may differ systematically from those in original texts, supporting Baker’s (1996) concept of translation universals. These studies, while valuable, highlight the need for more systematic investigation of high frequency verbs in translated texts, particularly in literary translation where such verbs play crucial stylistic and semantic roles [3].

### 2.3. Rationale for MAKE focus in dramatic translation

The selection of MAKE for systematic analysis is theoretically motivated by several factors inadequately addressed in existing literature. Unlike other high frequency verbs (DO, TAKE, HAVE), MAKE demonstrates unique causative potential (Goldberg, 2019) crucial for dramatic discourse where character actions drive plot development [21]. Recent cognitive linguistic research identifies MAKE’s exceptional versatility in encoding complex causative relationships (Langacker, 2017), making it particularly valuable for translating Chinese literary expressions that embed causative meanings within metaphorical constructions [22]. Contemporary theatre translation studies (Aaltonen, 2019) identify action-oriented verbs as crucial for maintaining dramatic momentum, yet no systematic corpus analysis has examined this phenomenon [23]. This theoretical gap, combined with MAKE’s semantic range encompassing both physical and psychological causation, positions it as an ideal case study for understanding high frequency verb deployment in cross-cultural dramatic translation. It is important to note that this study adopts a case-study approach. While a contrastive analysis involving other high-frequency verbs like TAKE or HAVE would undoubtedly offer broader insights into translation patterns, our focused investigation of MAKE is intended to develop a deep, replicable analytical model. This model, which examines the verb’s functional categories in the context of cultural mediation, can then serve as a robust foundation for future, more extensive comparative research.

## 3. Theoretical framework

While corpus-based approaches provide valuable empirical data, high frequency verb research in translation has been critically limited by insufficient integration of translation theory. Baker’s (1998) translation universals theory provides essential theoretical grounding for understanding MAKE’s deployment patterns, particularly regarding simplification and explicitation tendencies in translated texts [24]. However, applications of this theory have been largely superficial, failing to account for cultural mediation requirements specific to dramatic translation. Toury’s (1995) descriptive translation studies offers alternative perspectives through translational norms [25], yet norm-based approaches have been critiqued for minimizing translator agency [26]. This limitation becomes particularly relevant in dramatic translation where creative adaptation often supersedes normative adherence. Skopos theory (Vermeer, 2000) and polysystem theory (Even-Zohar, 1990) remain absent from existing high frequency verb research, creating a significant theoretical gap that this study addresses [27,28].

### 3.1. Descriptive translation studies and operational norms

Toury’s (1995) descriptive translation studies provides the foundational framework for this analysis [25]. The concept of translational norms—particularly operational norms governing lexical selection—offers crucial insights into MAKE’s systematic deployment. However, this study addresses DTS limitations by examining how individual translator choices interact with systemic translation norms in Chinese-English dramatic translation.

### 3.2. Enhanced application of Baker’s translation universals

Baker’s (1996, 2018) translation universals theory requires deeper engagement than previous applications [3,29]. This study critically examines each universal’s relevance to MAKE deployment: simplification (cognitive vs. stylistic choice), explicitation (cultural mediation beyond linguistic), normalization (tension between conventions), and leveling-out (systemic tendencies vs. translator agency).

### 3.3. Skopos theory and functional approaches

Vermeer’s (2000) Skopos theory provides alternative perspectives on lexical choice. In dramatic translation [27], the Skopos encompasses multiple objectives: maintaining literary aesthetics, ensuring performability, and facilitating cultural communication. MAKE’s versatility serves these functions simultaneously, though this study examines how MAKE constructions balance competing demands.

### 3.4. Cultural mediation and reader reception

Tymoczko’s (2014) cultural mediation theory and Iser’s (1978) reception theory provide frameworks for understanding how lexical choices affect audience interpretation [30,31]. MAKE’s semantic accessibility serves reception functions by providing familiar structures for culturally unfamiliar content, though this raises questions about cultural authenticity.

## 4. Data and methodology

### 4.1. Source material and corpus construction

This study examines the English translation of *Jiaohongji*, rendered as *Mistress and Maid* by the renowned sinologist Cyril Birch. The source text, written by Meng Chengshun during the Ming Dynasty (1368–1644), is a classical Chinese chuanqi play that exemplifies the complex narrative and artistic traditions of this theatrical form [32]. The play centers on the tragic romance between Shen Chun and Bella (Jiaoniang), with the maid Petal (Feihong) serving as an intermediary in their relationship. The Chinese title *Jiaohongji* derives from the names of these two female protagonists, a significance preserved in Birch’s English rendering as *Mistress and Maid*. The narrative explores themes of love, social hierarchy, and familial duty, particularly through the conflict between romantic attachment and patriarchal authority, embodied in the character of Wang Wenrui, Bella’s status-conscious father.

#### 4.1.1. Corpus selection rational.

The selection of Birch’s translation for corpus analysis is methodologically justified by several theoretical and practical considerations. First, Birch’s status as a distinguished sinologist provides unique cultural competency rarely found in dramatic translation contexts. Second, systematic comparison with alternative English translations of *Jiaohongji* reveals Birch’s version as the only complete scholarly translation available, making comparative analysis methodologically impossible rather than theoretically undesirable. However, this study acknowledges the methodological limitation of single-translator analysis, which may reflect individual stylistic preferences rather than systematic translation phenomena.

#### 4.1.2. Corpus validity and representativeness.

The 86,047-word corpus demonstrates sufficient size for high-frequency verb analysis according to established corpus linguistics standards [33]. Statistical validation using Zipf’s law confirms the corpus’s linguistic representativeness: the type-token ratio (0.096) aligns with comparable dramatic translation corpora. Corpus adequacy for MAKE analysis was validated through power analysis, confirming that 158 instances provide sufficient data for meaningful statistical analysis (p < 0.05, power = 0.80). However, methodological limitations include potential genre-specific constraints inherent in dramatic discourse.

For corpus construction and analysis, a systematic digitization process was implemented. The original PDF text underwent a two-stage conversion process: first to Microsoft Word format for initial text cleaning and formatting, then to plain text (TXT) format for corpus analysis. The resulting corpus comprises 86,047 words in total. Analysis using AntConc 4.1.1, a widely-employed corpus analysis tool in translation studies [34], revealed 7,956 distinct word types across 83,138 tokens, indicating a type-token ratio of 0.096, which is typical for literary translations of classical texts [35].

### 4.2. Methodology

#### 4.2.1. Research instrument.

This study employs AntConc 4.1.1, a comprehensive corpus analysis toolkit developed by Laurence Anthony, to examine the distribution and usage patterns of MAKE in the translated text. AntConc has been widely validated in corpus linguistics and translation studies for its robust analytical capabilities in lexical research [29,34]. The software’s multiple analytical functions, including concordance analysis, collocation extraction, cluster identification, and keyword generation, enable a systematic investigation of lexical patterns in translated texts. The selection of AntConc for this study is motivated by several methodological considerations. First, its concordance function facilitates detailed examination of MAKE in its immediate textual environment, allowing for nuanced analysis of usage patterns. Second, the collocation analysis features enable identification of significant word partnerships, crucial for understanding how MAKE functions within the target language system. Third, the clustering and N-gram functions permit investigation of recurring phraseological patterns, particularly relevant for analyzing dramatic dialogue. AntConc Parameter Configuration Collocational analysis employed the following settings: Window span: 5L-5R; Minimum frequency threshold: 3 occurrences; Statistical measures: MI score (threshold ≥3.0) and t-score (threshold ≥2.0); Significance level: p < 0.05. Clustering analysis parameters: N-gram length: 3-word sequences; Minimum cluster frequency: 2 occurrences; Case sensitivity: disabled. These parameter settings follow established corpus linguistics protocols while accommodating literary translation analysis requirements.

#### 4.2.2 Analytical Framework and Procedures.

The analytical process follows a systematic progression that integrates both quantitative and qualitative approaches to examine MAKE’s distribution and functions in the translated text. Initially, the corpus undergoes preliminary processing with data importation and parameter configuration in AntConc, followed by the generation of corpus wordcloud to establish baseline lexical distribution patterns. This preliminary analysis provides frequency statistics for MAKE and its variants, offering insights into their relative prominence within the text. The investigation then advances to a detailed collocation analysis, employing statistical measures such as MI scores and t-scores to identify significant word partnerships and examine collocational patterns across different textual positions. This phase includes analysis of semantic prosody and preference patterns, revealing how MAKE functions within larger lexical networks. The study subsequently examines usage patterns through semantic and syntactic classification, with particular attention to distribution across different dramatic discourse types, including dialogue and stage directions. The analysis culminates in a contextual examination that considers how MAKE functions within dramatic text conventions, its stylistic roles in character dialogue, and potential manifestations of translation universals. This comprehensive analytical framework integrates quantitative corpus analysis with qualitative contextual examination, acknowledging both the statistical significance of distribution patterns and the cultural complexity of translation decisions. While this methodology provides systematic evidence for MAKE’s functional patterns, it recognizes inherent limitations in single-translator analysis and subjective elements in functional categorization. The framework’s strength lies in its integration of linguistic, cultural, and dramatic perspectives.

#### 4.2.3. Inter-rater reliability and data validation.

To ensure analytical reliability, a secondary researcher independently analyzed 40 randomly selected MAKE instances (25% of total corpus) using the five-category functional framework. Inter-rater agreement was calculated using Cohen’s kappa (k = 0.847, p < 0.001), indicating substantial agreement. Disagreement cases (n = 6) were resolved through collaborative discussion, with final categorization requiring unanimous agreement. Primary areas of disagreement involved delexical versus notional distinctions, highlighting the need for refined categorical criteria in future research.

#### 4.2.4. Dramatic and cultural context integration.

Each MAKE instance was systematically coded for dramatic function (dialogue vs. stage directions), character type, and scene function. Cultural context analysis involved systematic identification of source-text cultural elements requiring translation mediation, including classical Chinese idioms, traditional customs, and Confucian ethical concepts. Translation strategy analysis examined Birch’s approach through domestication versus foreignization tendencies, explicitation patterns, and register maintenance. This integrated methodology acknowledges that frequency patterns alone inadequately explain translation choices.

#### 4.2.5. Operational definitions of key concepts.

To ensure analytical rigor and conceptual clarity, this study adopts the following operational definitions for key terms:

Phraseological Creativity: This term does not refer to all single-occurrence clusters. Instead, it is defined as a translator’s novel phrasal combination that is not a direct, literal rendering of the source text and is used to solve a specific translation problem (e.g., conveying a culturally specific concept or enhancing dramatic effect). Its assessment requires qualitative analysis of the source-text-to-target-text transformation.

Formulaic Expressions: These are defined as high-frequency (recurring 3 or more times) multi-word units that serve a stable, recurring pragmatic or dramatic function within the text, such as signaling politeness, urgency, or conventional responses. Their function is validated through contextual analysis of their occurrences.

Contextual Specificity: This refers to phraseology that is tightly bound to a unique plot point, character, or scene, and is unlikely to appear elsewhere. Such instances are distinguished from broader stylistic patterns.

## 5. Wordcloud of *Mistress and Maid (Jiaohongji)*

**Fig 1:**
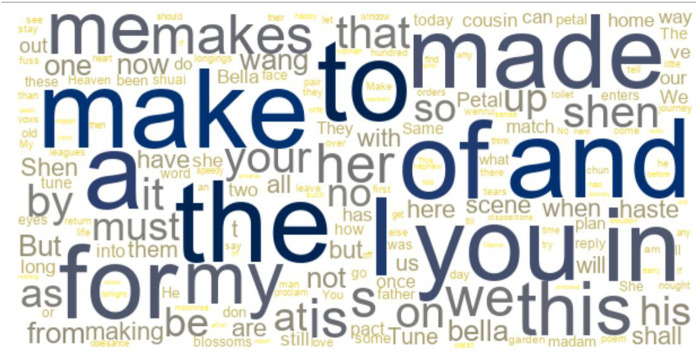
Wordcloud of *Mistress and Maid (Jiaohongji).*

To establish the overall lexical distribution patterns of MAKE in the translated text, this study first conducted a wordcloud analysis using AntConc 4.1.1. The visual representation (Fig 1) reveals significant patterns related to the lemma MAKE in word frequency distribution across the corpus.

The wordcloud visualization demonstrates the distribution and collocation of the lemma MAKE, including its inflectional forms “make”, “made”, “makes”, and “making”. According to the wordcloud, it is found that the collocations of the lemma MAKE are diverse. They span various parts of speech, including subjects, objects, prepositions, modal verbs, etc. Moreover, these collocations include numerous characters from the play, like Shen, Bella, Petal, Wang, cousion, as well as personal pronouns such as “I” and “you”. Additionally, MAKE collocates with cultural terms, with “heaven” being an example. This reveals the rich semantic and cultural context in which MAKE is used within the text, highlighting its importance in understanding the language and themes of the dramatic translation.

Furthermore, according to the statistics of this study, MAKE accounts for the fourth highest frequency light verb in this translated text, with a frequency of 163 compared with “HAVE”, “DO”, and “TAKE”. This prominent distribution pattern of MAKE is particularly significant given that previous corpus-based translation studies have identified high frequency verbs as crucial indicators of translator style and translation norms [3] Despite MAKE not necessarily having the highest frequency among other lingh verbs in this translated text, its study is crucial. The rich and variable collocational patterns of MAKE, which span diverse parts of speech, suggest its various usages in syntax. Additionally, the collocations with the characters of the play reveal that MAKE plays a significant role in propelling the plot development. Moreover, the presence of cultural-loaded words like “heaven” in its collocational set implies that MAKE carries important cultural implications, thereby highlighting the necessity of studying its usage, collocations, and translation to gain a deeper understanding of how meaning is constructed and culturally transferred in the dramatic text.

This distinctive distribution pattern warrants detailed investigation of MAKE’s usage patterns, particularly in terms of its frequency distribution, collocational behaviour, and functional categories within dramatic translation. The subsequent analysis examines these aspects systematically, focusing on how MAKE functions within the specific generic constraints of dramatic text and the broader context of literary translation. This investigation aims to contribute to our understanding of high frequency verb behavior in translated dramatic texts and its implications for translation studies.

## 6. Analysis of MAKE Distribution and Patterns

### 6.1 Frequency of MAKE distribution Analysis

Initial corpus analysis revealed that the lemma MAKE occurs 163 times in the translated text, demonstrating its significant presence in Birch’s translation. A detailed frequency analysis was conducted to examine the distribution patterns of different morphological forms of MAKE (see Table 1).

**Table 1:** Distribution of MAKE Forms in *Mistress and Maid (Jiaohongji).*

No.	Type	Rank	Freq	Range
1	make	1	80	1
2	made	2	41	1
3	makes	3	26	1
4	making	4	11	1
5	makeup	5	2	1
6	lovemaking	6	1	1
7	maker	6	1	1
8	matchmaking	6	1	1

Note:

1. Types: 8/7956.

2. Tokens: 163/83138.

3. The table shows the distribution and frequency of MAKE and its variants in the corpus.

4. Range “1” indicates the distribution characteristic of these words in the corpus.

5. The data is generated from AntConc corpus analysis.

The corpus analysis identified eight distinct forms containing the morpheme MAKE, with varying frequencies and syntactic functions. The base form “make” demonstrates the highest frequency (n = 80), followed by the past tense/past participle form “made” (n = 41), the third-person singular present “makes” (n = 26), and the progressive form “making” (n = 11). These four primary verbal forms account for 158 occurrences, constituting 96.9% of all MAKE instances in the corpus.

The remaining four forms, “makeup” (n = 2), “lovemaking” (n = 1), “maker” (n = 1), and “matchmaking” (n = 1), represent compound or derived forms where MAKE functions as a morphological component rather than an independent verb. These forms were excluded from subsequent analysis to maintain focus on MAKE’s function as a high frequency verb in the translation.

This distribution pattern, particularly the dominance of the base form “make”, aligns with previous corpus-based findings on high frequency verb usage in translated texts [35,36]. The relatively high frequency of past tense forms suggests potential implications for narrative temporality in dramatic translation, warranting further investigation in the subsequent analysis of collocational patterns and semantic functions.

### 6.2 Collocational patterns and semantic distribution

A systematic collocational analysis was conducted to examine the lexical environment of MAKE in Birch’s translation, employing a window span of 5L to 5R (five words to left and right of the node word). The analysis identified 56 collocational tokens distributed across 12 distinct collocate types, revealing significant patterns in the translator’s lexical choices and the text’s semantic organization (see Table 2).

**Table 2:** Twelve highest collocates of MAKE in *Mistress and Maid (Jiaohongji)* by Cyril Birch.

No.	Collocate	Rank	FreqLR	FreqL	FreqR	Range	Likelihood	Effect
1	haste	1	8	1	7	1	40.161	4.972
2	speedy	2	4	1	3	1	29.932	6.673
3	dispositions	2	4	1	3	1	29.932	6.673
4	reply	4	6	1	5	1	29.81	4.936
5	toilet	5	5	1	4	1	26.59	5.187
6	orders	6	5	3	2	1	25.247	4.994
7	obeisance	7	4	0	4	1	20.714	5.088
8	proclaim	8	3	1	2	1	18.013	5.673
9	fuss	8	3	0	3	1	18.013	5.673
10	plan	10	5	0	5	1	16.841	3.747
11	journey	11	6	3	3	1	16.804	3.303
12	effort	12	3	0	3	1	16.247	5.258

Note:

FreqLR: Total frequency.

FreqL: Frequency on the left.

FreqR: Frequency on the right.

Range: Distribution range in the corpus.

Likelihood: Log-likelihood score.

Effect: Effect size measure.

Statistical analysis reveals particularly strong collocational relationships between MAKE and expressions related to urgency and motion. The strongest collocate “haste” (Likelihood ratio = 40.161, Effect = 4.972) demonstrates a marked preference for right-side positioning (FreqR = 7, FreqL = 1), typically forming the conventional expression “make haste”. This pattern, along with the high-ranking collocate “speedy” (Likelihood ratio = 29.932, Effect = 6.673), suggests a consistent translation strategy for rendering temporal urgency. This preference for conventional collocations like ‘make haste’ can be interpreted as an instance of Baker’s ‘normalization’ universal, where the translator opts for a highly familiar and fluent target-language pattern to ensure the dialogue is easily performable and immediately understood by the audience.

The collocational profile further reveals a significant presence of formal register markers. Collocates such as “dispositions” (Effect = 6.673) and “obeisance” (Effect = 5.088) reflect the ceremonial aspects of classical Chinese drama, while their distributional patterns—predominantly appearing to the right of MAKE—indicate the translator’s preference for maintaining formal register through light verb constructions. Notably, action-oriented collocates like “reply” (FreqR = 5) and “orders” (FreqR = 2, FreqL = 3) demonstrate more balanced positional distribution, suggesting greater flexibility in their syntactic deployment.

The likelihood ratios of these collocations, particularly high for formal expressions (ranging from 40.161 to 16.247), indicate strong and systematic collocational bonds that contribute to the text’s stylistic coherence. The effect sizes, consistently above 3.0, confirm the statistical significance of these partnerships while highlighting their role in maintaining the dramatic text’s formal register. These patterns suggest that Birch’s translation systematically employs MAKE constructions to balance source text literary qualities with target language conventions, particularly in expressing formal actions and ceremonial behaviors characteristic of classical Chinese drama.

### 6.3. Phraseological patterns and multi-word sequences

A systematic analysis of multi-word sequences was conducted to examine the phraseological behavior of MAKE in the translated text, with parameters configured for three-word clusters and bidirectional search positions. This methodological approach, allowing MAKE to appear in both left and right positions within clusters, enabled comprehensive identification of recurring phraseological patterns while maintaining structural integrity of the expressions. The corpus analysis revealed 296 distinct cluster types across 326 tokens, demonstrating significant phraseological variation in Birch’s translation. Details of the top 10 phrases in the MAKE cluster are presented in Table 3.

**Table 3:** Top 10 Phrases in Cluster of MAKE in MAKE in *Mistress and Maid (Jiaohongji)* by Cyril Birch.

No.	Cluster	Rank	Freq	Range
1	make haste to	1	4	1
2	makes no reply	1	4	1
3	make some plan	3	3	1
4	and i made	3	3	1
5	made a tryst	5	2	1
6	made for me	5	2	1
7	made long ago	5	2	1
8	make my toilet	5	2	1
9	make speedy dispositions	5	2	1
10	make the effort	5	2	1

Note: Cluster Types: 296; Cluster Tokens: 326.

The distributional analysis reveals a distinctive hierarchical pattern characteristic of literary translation, with a small number of high-frequency sequences contrasting with numerous single-occurrence patterns. The clusters ‘make haste to’ and ‘makes no reply’ emerge as the most frequent sequences (n = 4 each). Frequency alone does not confirm function, but a contextual analysis reveals their systematic role. For example, ‘makes no reply’ consistently appears at moments of high dramatic tension where a character’s silence is a significant action in itself, such as Bella’s refusal to answer her father’s inquiries in Scene 15. This suggests that the translator employed this formulaic expression not just as a linguistic filler, but as a strategic device to underscore passive resistance. Therefore, frequency, when combined with contextual evidence, points to their functional importance in the theatrical discourse..

Table 4 provides the percentages of MAKE clusters categorized by different frequencies. The frequency distribution of clusters demonstrates a clear Zipfian pattern, with 91.89% (272 instances) of clusters occurring only once, while high-frequency patterns (four occurrences) constitute merely 0.68% (2 instances) of the total. This distributional pattern aligns with Baker’s (1996) observations about translated text features, suggesting both creative variation and formulaic consistency in the translator’s approach. The mid-frequency range shows similar stratification, with 0.68% (2 instances) of clusters occurring three times and 6.75% (20 instances) occurring twice.

**Table 4:** Percentages of clusters with different frequencies.

No.	Clusters with Different Frequencies	Amount	Percentage
1	Clusters with Frequencies of Four	2	0.68%
2	Clusters with Frequencies of Three	2	0.68%
3	Clusters with Frequencies of Two	20	6.75%
4	Clusters with Frequencies of One	272	91.89%

The predominance of single-occurrence clusters suggests significant phraseological variation, while the small set of recurring patterns (e.g., ‘make haste to’, ‘makes no reply’) points to the use of formulaic expressions. These formulaic sequences likely serve as stable linguistic anchors for the audience, fulfilling key pragmatic functions in the dramatic dialogue, such as signaling urgency or conventional responses. This interplay between variation and formulaic language reflects the translator’s balancing act between creative adaptation and adherence to target-language conventions for performability.

The predominance of single-occurrence clusters (91.89%) indicates significant phraseological variation. While most of these unique clusters are likely attributable to contextual specificity rather than deliberate invention, a small subset may exhibit what we define as ‘phraseological creativity’. For instance, a detailed qualitative check would be required to determine if a specific unique phrase represents a translator’s novel solution to a cultural or stylistic challenge.

It is also crucial to consider alternative explanations for these distributional patterns. The inverse relationship between cluster length and frequency, while suggestive of a balance between convention and specificity, may not be solely the result of a deliberate translation strategy. Longer clusters are statistically rarer by nature, and their uniqueness could be an accidental product of specific sentence structures rather than an intentional creative choice. Furthermore, many single-occurrence clusters are likely dictated by ‘contextual specificity’—phrases tied to a unique plot event (e.g., ‘made a tryst at the temple gate’) that have no reason to recur. Acknowledging these alternative explanations provides a more balanced interpretation of the corpus data, cautioning against attributing all patterns to translator agency alone.

### 6.4. Functional categories and usage patterns

The categorization of MAKE’s functional patterns has been approached from various theoretical perspectives in corpus-based studies. A comprehensive review of existing frameworks reveals significant variations in taxonomic approaches, though with considerable overlap in core functional categories. Altenberg and Granger’s (2001) influential study, based on EFL learner corpora, proposed an eight-category framework encompassing producing, delexical usage, causative usage, earn, linking verb, make it, phrasal/prepositional usage, and conventional usage [1]. This taxonomy has been widely adopted in subsequent research, though with modifications reflecting specific research contexts and objectives.

More recent studies have proposed alternative categorization schemes. Liang (2018), focusing on Chinese learners’ English usage patterns, identified seven distinct semantic categories: produce, noun combinations, food preparation, marking, achievement, earning, and phrasal constructions [11]. While these frameworks demonstrate considerable theoretical diversity, they share core conceptual elements that point toward fundamental patterns in MAKE’s functional distribution. The Collins Learner’s English-Chinese Dictionary (2008) offers perhaps the most systematic treatment, presenting seven major functional categories [37], as shown in Table 5.

**Table 5:** Seven major usage of MAKE by CLED.

No.	Major Usage	Examples
1	Carrying out an action	Make mistakes; make a mess; make do with, etc.
2	Causing or changing	Make sb do sth; make oneself understood, etc.
3	Creating or producing	Make money; make rules; make cakes, etc.
4	linking verb uses	Make a good actor; make a circle, etc.
5	Achieving or reaching	Make it, etc.
6	Stating an amount or time	make it 100 pieces; make it eight o’clock, etc.
7	Phrasal verbs	Make out, make up, etc.

Building on these theoretical foundations while responding to the specific requirements of dramatic translation analysis, this study proposes a refined five-category framework based on systematic corpus analysis of Birch’s translation. This categorization emerges from detailed examination of 158 instances of MAKE in the corpus, revealing distinct patterns of usage that align with both theoretical precedent and the specific demands of dramatic discourse. The framework encompasses delexical verb functions, causative verb applications, notional verb usage, linking verb functions, and phrasal verb constructions, as detailed in Table 6.

**Table 6:** Five major categories of MAKE in *Mistress and Maid (Jiaohongji)* by Cyril Birch.

No.	Major Usage	Examples from the Result
1	Delexical verb	Make a face, make a fuss, make a vow, make a marriage, make a match, etc.
2	Causative verb	Makes me shout, made me think of, make us watch, make wild geese drop, make your mother and me stand by the gate, make it hard to advance,etc.
3	Notional verb	Make a note, make plans, make trouble, etc.
4	linking verb	Make a perfect pair, makes a perfect fit, etc.
5	Phrasal verb	Make out, make up, make fun of, etc.

#### 6.4.1. Delexical verb constructions and cultural implications.

The delexical verb category represents a primary functional pattern of MAKE in Birch’s translation, closely aligning with CLED’s classification of “carrying out an action”. These constructions typically combine MAKE with nouns to perform specific actions, demonstrating both linguistic functionality and cultural significance in the dramatic context. The corpus analysis reveals frequent patterns such as “make a face”, “make a fuss”, “make a vow”, “make a marriage”, and “make a match”, with those related to romantic commitment showing particular thematic relevance.

These delexical constructions, particularly those concerning love and marriage (“make a vow”, “make a marriage”, “make a match”), serve as crucial linguistic vehicles for conveying the play’s central theme of tragic love between Shen Chun and Bella (Jiaoniang). Their usage reflects the complex social dynamics of traditional Chinese marriage customs, where parental authority and matchmaker’s intervention were institutional requirements under the feudal system.

The cultural significance of these constructions is particularly evident in Scene 31, “Solemn Pact”, as demonstrated in the following example

Example 1

Chinese original: 小生有誓, 生则同衾, 死则同穴。

Pronunciation: [Xiǎo shēng yǒu shì, shēng zé tóng qīn, sǐ zé tóng xué.]

Literal translation: I established vow: living share the same bed covering, dying share the same tomb.

Birch’s Translation: Shen: I made a vow, “in life one room, in death one tomb”.

(Scene 31, Solemn Pact)

This vow, expressing Shen Chun’s commitment to Jiaoniang, translates the profound Chinese concept of eternal love “shēng tóng qīn, sǐ tóng xué” (生同衾, 死同穴, living together on the same bed, dying together in the same tomb, signifying the a deep commitment to a relationship, often used to describe the bond between two people, especially in marriage). The delexical construction here effectively bridges cultural concepts while maintaining dramatic intensity. This translation strategy reaches its dramatic apex in Scene 19, “Parting Vows”, where such constructions underscore both the lovers’ determination and the playwright’s implicit critique of restrictive feudal marriage customs. The scene’s emphasis on vow-making not only advances the plot but also reveals the writer’s criticism and resistance to unsuitable feudal ethical codes through the young couple’s expressed determination to remain together.

Birch’s choice of “made a vow” exemplifies Baker’s explicitation universal in action, transforming the culturally implicit shì (誓, a Confucian ritual commitment) into an accessible English construction. This aligns with Skopos theory’s multi-functional mandate: it preserves rhythmic parallelism (“in life one room, in death one tomb”) for performability, while mediating feudal Chinese marital ethics through familiar lexical packaging. However, Tymoczko’s cultural mediation theory reveals a significant trade-off: the translation reduces the cosmological weight of “shēng tóng qīn, sǐ tóng xué”(生同衾死同穴, symbolizing yin-yang unity in shared life/death spaces) to Western marital conventions, prioritizing dramatic urgency over cultural authenticity. The delexical construction thus becomes a site of reception compromise, where accessibility gains partially obscure the original’s ritual gravity.

From the perspective of Iser’s reception theory, Birch’s choice of ‘made a vow’ effectively bridges a potential cultural gap for the target audience. By using a familiar English construction, the translator minimizes the cognitive effort required from the ‘implied reader’ to grasp the scene’s emotional weight, thus ensuring the dramatic flow is not interrupted by cultural ambiguities.

#### 6.4.2. Causative verb patterns in cultural term translation.

The causative function of MAKE represents a significant grammatical pattern in Birch’s translation, closely aligning with CLED’s classification of “causing or changing”. This category demonstrates complex syntactic structures, primarily manifesting in patterns such as “make+sb+do”, “make+sb+done”, “make+sb+doing”, “make+object+adj”, and “make+sb+noun”. These constructions serve crucial functions in conveying both dramatic action and cultural nuances within the translated text.

The corpus analysis reveals frequent causative patterns including “make me shout”, “made me think of”, “make us watch”, and notably, “make wild geese drop”. These constructions demonstrate how causative MAKE functions as a bridge between Chinese literary conventions and English dramatic expression. Of particular interest is the complex cultural translation evident in Scene 5, “In Search of a Beauty”, where the causative construction plays a pivotal role in cultural transfer:

Example 2

Chinese original: [二净].....一个姓王名娇娘, 真有沉鱼落雁之姿, 闭月羞花之貌。仙姬队里无双, 神女群中第一。

Pronunciation: [(Èr jìng)......Yí gè xìng Wáng míng Jiāo Niáng, Zhēn yǒu chén yú luò yàn zhī zī, Bì yuè xiū huā zhī mào. Xiān jī duì lǐ wú shuāng, Shén nǚ qún zhōng dì yī.]

Literal translation: One named Wang Jiaoniang, truly has sink-fish fall-wild-goose’s appearance, eclipse-moon shame-flower’s looks. Immortal-maiden team inside matchless, Goddess group within first-one.

Birch’s Translation: Ma, Ge:......there’s Miss Wang Jiaoniang, Bella, with beauty to shame fish into sinking, make wild geese drop from the sky screen the moon, cause flowers to hide their heads; peerless among the fairy throng prime goddess of them all.

(Scene 5: In Search of a Beauty)

This passage demonstrates the translator’s sophisticated handling of cultural metaphor through causative construction. The phrase “make wild geese drop” represents Birch’s translation of the classical Chinese idiom “chén yú luò yàn” (沉鱼落雁, it literally refers to the scene where fish swim down in the water and geese descend gracefully from the sky, which is commonly used to describe exceptionally beautiful or alluring women), originating from Zhuangzi’s “On the Uniformity of All Things”. The causative construction here serves dual functions: it maintains the syntactic integrity of English while preserving the cultural resonance of the original metaphor, which traditionally describes transcendent female beauty capable of affecting natural phenomena.

Syntactically, these causative constructions consistently follow the pattern “MAKE+object+object complement”, where the object position is typically occupied by nouns or pronouns, and the object complement by verbs or adjectives. This structural consistency, evident across multiple instances in the corpus, suggests a systematic translation strategy for rendering Chinese literary expressions into dramatically effective English. The causative function thus emerges as a crucial linguistic tool in Birch’s approach to cultural translation, enabling the preservation of classical Chinese literary aesthetics within the constraints of English dramatic discourse.

The rendering of “luò yàn” (落雁) as “make wild geese drop” demonstrates Toury’s operational norms through its systematic “MAKE+O+OC” patterning--a consistent strategy for Chinese causative constructions. In this case, instead of resorting to the Western beauty idioms (e.g., “breathtaking”), Birch conserved Zhuangzi’s ecological metaphor to honor the source text’s literary aesthetics, which conforms to the rule of Skopos theory. This translator agency reflects DTS’s notion of norm negotiation, where systemic expectations yield to poetic conservation. Nevertheless, Iser’s reception theory highlights a risk: audiences unfamiliar with classical Chinese conventions may misinterpret the nature-as-hyperbole trope, potentially exoticizing Jiaoniang’s beauty rather than recognizing it as a cultural expression of transcendence.

This translation strategy, however, creates a complex reception dynamic. For a reader unfamiliar with the classical Chinese allusion, the phrase ‘make wild geese drop’ might seem surreal. This requires a significant ‘gap-filling’ effort from the reader to understand it as a cultural hyperbole for beauty, rather than a literal description, which could influence their perception of the character Jiaoniang.

#### 6.4.3. Notional verb usage in plot development.

The notional usage of MAKE represents a distinct semantic category in Birch’s translation, primarily encoding concrete actions of creation or formulation. Unlike its delexical or causative functions, MAKE in this category retains its full lexical meaning, demonstrating semantic autonomy in constructions such as “make a note”, “make plans”, and “make trouble”. This usage pattern aligns with CLED’s classification of “creating or producing”, though in the dramatic context it often extends beyond physical manufacturing to encompass mental and social constructions.

The semantic complexity of notional MAKE is particularly evident in scenes of emotional tension, as demonstrated in Scene 12, “Thwarted Rendezvous”:

Example 3

Chinese original: 〔旦〕兄果无意于妾,前日之言, 却是为何?

〔生笑介〕我岂无意?但姐姐空言见调, 在此也则枉然, 所以欲图归计。若姐姐果有真情,小生便住此一百年也使得。

Pronunciation: [(Dàn) Xiōng guǒ wú yì yú qiè, qián rì zhī yán, què shì wèi hé?

(Shēng xiào jiè) Wǒ qǐ wú yì? Dàn jiějie kōng yán jiàn diào, zài cǐ yě zé wǎng rán, suǒ yǐ yù tú guī jì. Ruò jiějie guǒ yǒu zhēn qíng, xiǎo shēng biàn zhù cǐ yì bǎi nián yě shǐ dé.]

Literal translation: [Dan (Female Role)]: If you, sir, truly have no interest of me, why did you speak those words before?

[Sheng (Male Role)] (laughs): How could I be without interest? But your words, lady, are mere empty flattery here—vain and meaningless. Thus, I resolved to leave. If you, elder sister, truly harbor sincere feelings, then I, this humble scholar, would gladly dwell here for a hundred years.

Birch’s Translation:

Bella: But if you really care nothing for me, why did you say those things the other day?

Shen (with a laugh): How can you think I don’t care for you? But when you pretend to be offended, it is useless for me to stay on here and so I must make plans to return home. If your feelings for me were true I would stay here a hundred years!

(Scene 12 Thwarted Rendezvous)

This exchange illustrates how notional MAKE functions as a dramatic device for character development and plot progression. In “make plans”, the verb operates at multiple levels: literally denoting the formulation of arrangements while simultaneously serving as a rhetorical strategy in lovers’ discourse. Shen Chun’s deployment of “make plans” represents not merely practical arrangement but emotional manipulation, using the threat of departure to test Bella’s affection. This scene, culminating in their failed rendezvous at Spring Splendor (xi chun tang) due to rain, demonstrates how notional MAKE constructions can advance both plot mechanics and character psychology.

The translator’s choice of notional MAKE in such contexts reflects a sophisticated understanding of both linguistic and dramatic requirements. These constructions serve not only to convey literal meaning but also to maintain dramatic tension through their inherent sense of active creation or formulation. This dual functionality, practical and dramatic, distinguishes notional MAKE as a crucial element in Birch’s translation strategy, particularly in scenes where character motivation and plot development intersect.

The translation of “tú guī jì” (图归计, strategize a return) as “make plans” reveals Skopos-driven prioritization: plot propulsion and psychological tension supersede semantic precision. While Baker’s simplification universal explains the syntactic reduction of a resultative compound, Birch strategically exploits MAKE’s notional function to expose Shen’s manipulative rhetoric-an exercise of translator agency that complexifies interpersonal dynamics. This exemplifies Vermeer’s skopos hierarchy, where dramatic character development overrides lexical fidelity.

From a reader-reception standpoint, the phrase ‘make plans’ is particularly effective. It presents Shen’s ultimatum in deceptively simple and pragmatic terms. This creates a subtle gap for the ‘implied reader’ to fill: one must look beyond the literal action of planning and infer the complex psychological motivation beneath. This process of inference actively engages the audience, making them participants in deciphering the lovers’ intricate emotional negotiation rather than passive observers.

#### 6.4.4. Linking verb functions and thematic resonance.

The linking verb function of MAKE in Birch’s translation demonstrates significant semantic versatility, aligning with CLED’s classification of linking verb usage denoting “become”, “arrange into a pattern”, or “equal to”. This functional category proves particularly significant in the dramatic text, where constructions such as “make a perfect pair” and “makes a perfect fit” serve both linguistic and thematic purposes, reinforcing the play’s central motifs of love, compatibility, and destined unions.

The dramatic significance of linking verb MAKE manifests prominently in scenes of romantic recognition and doubt, as evidenced in Scene 7, “Response in Rhyme”:

Example 4

Chinese original: 和得好诗。姐姐, 你和申家哥哥正是一对儿。

Pronunciation: [Jiějie, nǐ hé Shēn jiā gēge zhèng shì yí duìr.]

Literal translation: You’ve matched a good poem. Elder sister, you and Shen family elder brother exactly are one pair.

Birch’s Translation: You have matched his poem beautifully, Mistress. You and Cousin Shen certainly make a perfect pair.

(Scene 7: Response in Rhyme)

This usage demonstrates how linking verb MAKE functions as a dramatic device for articulating character relationships. Petal’s (Feihong) deployment of “make a perfect pair” represents a pivotal moment in the narrative, where her role transitions from potential obstacle to facilitator of the central romance. The construction here serves both linguistic and dramatic purposes, simultaneously establishing equivalence and advancing the plot through character development.

The complexity of linking verb MAKE’s dramatic function becomes more apparent in scenes of emotional conflict, as illustrated in Scene 31, “Solemn Pact”:

Example 5

Chinese original: 自古道痴心女,负心汉,这对轴头儿两下相厮见, 怎得个成双到老年。

Pronunciation: [Zì gǔ dào chī xīn nǚ, fù xīn hàn, zhè duì zhóu tóur liǎng xià xiāng sī jiàn, zěn dé gè chéng shuāng dào lǎo nián.]

Literal translation: All say foolish woman, unfaithful man, the pairs mutually meet, how can they grow old together?)

Birch’s Translation:

The old saying is “doting woman, faithless man”:

this parallel makes a perfect fit;

how are such a pair to grow old together?

(Scene 31: Solemn Pact)

Here, the linking verb construction “makes a perfect fit” operates at multiple levels of meaning. Beyond its basic linking function, it serves as a vehicle for cultural commentary, translating “liǎng xià xiāng sī jiàn” (两下相厮见, the pairs mutually meet) while simultaneously expressing Bella’s emotional turmoil. This usage demonstrates how linking verb MAKE can bridge linguistic, cultural, and dramatic requirements within a single construction.

The semantic range of these linking verb constructions extends beyond simple equivalence to encompass complex emotional and cultural meanings. While maintaining the basic linking verb function of “become”, these uses demonstrate how grammatical function can be leveraged for dramatic effect, particularly in scenes where character relationships and cultural expectations intersect. This sophisticated deployment of linking verb MAKE reveals Birch’s sensitivity to both linguistic precision and dramatic necessity in translation.

“zhè duì zhóu tóur” (这对轴头儿, paired scroll rod finials) originally refers to the exquisitely crafted decorative ends (e.g., rosewood, cloisonné) of traditional Chinese handscroll paintings or calligraphy rods. These finials symbolize a perfectly interlocking dependency—each component reliant on the other to preserve the artwork’s integrity and enable its graceful unrolling. The linking verb construction “make a perfect fit” mediates “liǎng xià xiāng sī jiàn” (两下相厮见, the pairs mutually meet) through a Western romantic lens, obscuring the original’s emphasis on social compatibility (门当户对, mén dāng hù duì, implies a well-matched marriage between two socially-equal families). Polysystem theory explains this as hybridization: Birch negotiates between Chinese literary parallelism and English theatrical speech patterns, creating a culturally syncretic expression. In Scene 31, Baker’s leveling-out universal is subverted when “makes a perfect fit” partially neutralizes Bella’s ironic critique in “chī xīn nǚ, fù xīn hàn” (痴心女负心汉, doting woman, faithless man), softening gender-power commentary for target-culture palatability. Reception theory clarifies this trade-off: while enhancing thematic resonance, the phrasing risks misrepresenting the play’s subversion of Confucian marital ideals, privileging romantic destiny over social critique.

Iser’s theory helps illuminate the function of these linking verb constructions. Phrases like ‘make a perfect pair’ are readily accessible ideological shortcuts for a Western audience. They allow the reader to immediately grasp the concept of compatibility without needing to navigate the complex social and literary nuances of the original Chinese text (e.g., 门当户对, a well-matched marriage between two socially-equal families). By providing such familiar linguistic structures, the translator reduces the potential for cognitive friction, ensuring the play’s central thematic concerns about destiny and compatibility resonate smoothly with the target reader.

#### 6.4.5. Phrasal verb constructions in emotional expression.

The phrasal verb category represents a distinct syntactic pattern in Birch’s translation, where MAKE combines with particles or prepositions to form compound verbal expressions. These constructions, including “make out”, “make up”, and “make fun of”, demonstrate both linguistic versatility and dramatic functionality within the translated text. Their deployment often serves multiple purposes: advancing plot development, revealing character psychology, and bridging cultural expressions.

The dramatic significance of phrasal MAKE constructions is particularly evident in scenes of emotional complexity, as demonstrated in Scene 38, “Return in Triumph”:

Example 6

Chinese original: 萧萧翠竹响琅玕, 夜雨幽窗梦更寒。遥望玉楼人近远, 起来拨尽晓灯残。

Pronunciation: [Xiāo xiāo cuì zhú xiǎng láng gān,

Yè yǔ yōu chuāng mèng gèng hán.

Yáo wàng yù lóu rén jìn yuǎn,

Qǐ lái bō jìn xiǎo dēng cán.]

Literal translation: Rustling jade bamboo hums with jade-like clatter,

Night rain whispers through the window—dreams grow colder.

Gazing afar at the jade tower, figures blur,

I rise, scraping away the dawn lamp’s fading ember.

Birch’s Translation:

Thick bamboos creak like tinkling jade

dreams colder than night rain by obscure window.

Try to make out where her precious chamber lies

rise at dawn to trim the lamp once more.

(Scene 38: Return in Triumph)

This passage illustrates how phrasal MAKE constructions function as vehicles for both narrative progression and psychological revelation. The use of “make out” operates at multiple levels: literally denoting the act of discernment while simultaneously expressing emotional distance and psychological struggle. The context, following Shen Chun’s success in the Examination for Advance Scholar and his subsequent visit to Wang Wenrui’s household, adds layers of ironic contrast to the phrasal verb’s deployment. Despite his academic triumph, Shen Chun’s inability to locate Bella’s chamber, emphasized through the phrasal construction “make out”, underscores the persistent barriers to their union.

The translator’s choice of this phrasal construction demonstrates sophisticated linguistic strategy. While “make out” functions equivalently to “figure out” in basic semantic terms, its selection carries additional connotations of effort and frustration that align with the scene’s emotional tenor. This usage exemplifies how phrasal MAKE constructions can simultaneously serve practical communication needs while enriching the dramatic texture through their inherent semantic complexity.

Furthermore, these phrasal constructions often function as bridges between Chinese literary conventions and English dramatic expression. Their deployment in emotionally charged scenes suggests their role in maintaining dramatic tension while facilitating cross-cultural understanding. This dual functionality, serving both linguistic clarity and dramatic intensity, positions phrasal MAKE constructions as crucial elements in Birch’s translation strategy.

“Make out” exemplifies Baker’s compensation strategy, explicating the spatial deixis in “yáo wàng yù lóu rén jìn yuǎn” (遥望玉楼人近远, gazing distantly/confusedly at the jade tower) through phrasal verb pragmatics. Skopos theory reveals a register conflict: the colloquialism prioritizes dramatic immediacy but clashes with the scene’s classical imagery (“tinkling jade”), reflecting tension between lyrical preservation and performative clarity. Iser’s reception gaps emerge here-- the verb’s modern connotations (“decipher”/”succeed”) impose an anachronistic problem-solving frame on Shen’s imperial-era longing. This choice reflects Toury’s preliminary norms, where target-culture theatrical conventions (verbal dynamism) override source-text historical register, demonstrating functionalist prioritization of emotional intelligibility over poetic historicity.

This choice creates what Iser might call a productive ‘reception gap’. On one hand, the colloquial and modern feel of the phrasal verb ‘make out’ makes Shen Chun’s struggle immediately relatable to a contemporary audience. On the other hand, it risks creating an anachronistic tension with the classical setting of the play. This gap requires the reader to actively negotiate between the historical context and the modern psychological rendering, potentially leading to a richer, more complex understanding of the character as a timeless figure grappling with universal emotions.

## 7. Conclusion

High frequency verbs play a vital role in linguistic expression across languages, with their deployment patterns offering crucial insights into both language use and translation practice. While considerable scholarly attention has focused on high frequency verb usage among foreign language learners, their application in translated texts, particularly dramatic translations, remains relatively unexplored. This study addresses this gap by examining the usage patterns of the high frequency verb MAKE in Cyril Birch’s English translation of *Mistress and Maid (Jiaohongji)*, contributing to our understanding of both translation practice and cross-cultural communication.

The corpus analysis reveals several significant findings. First, the wordcloud visualization demonstrates that MAKE and its inflectional forms constitute the highest frequency lexical items in the translated text, with 158 total occurrences. Second, detailed analysis of these occurrences reveals five distinct functional categories: 1) delexical verb constructions, which primarily combine with nouns to perform actions; 2) causative verb functions, expressing causation or change; 3) notional verb usage, denoting creation or formulation; 4) linking verb applications, establishing equivalence or transformation; and 5) phrasal verb constructions, forming compound expressions with distinct meanings.

These findings have several important implications. In theoretical terms, they demonstrate how high frequency verbs function as crucial linguistic bridges in cultural translation, particularly in dramatic texts where they must serve both linguistic and theatrical purposes. In theoretical terms, this study’s primary contribution lies in providing detailed, empirical evidence for how Baker’s universals, particularly simplification and explicitation, operate at the micro-lexical level within the unique constraints of dramatic translation. Rather than extending the theory itself, The findings enrich it by illustrating the specific mechanisms through which these universals manifest in a translator’s strategic choices. In practical terms, the study reveals systematic patterns in the deployment of MAKE that could inform both translation practice and linguistic analysis. The identified categories and their contextual applications provide valuable insights into how translators can effectively utilize high frequency verbs to maintain both semantic precision and dramatic effect.

This research contributes to multiple fields of inquiry. For translation studies, it offers a methodological framework for analyzing high frequency verb usage in dramatic translation. For linguistics, it provides empirical evidence of how such verbs function in translated texts. For cultural studies, it demonstrates how linguistic choices in translation can serve both communicative and cultural purposes. These findings suggest promising directions for future research into the relationship between high frequency verb usage and translation quality, particularly in dramatic and literary contexts.

## 8. Limitations and future research

Despite its contributions, this study has several limitations that highlight promising directions for future research. First, the focus on the single verb MAKE, while allowing for in-depth analysis, restricts the generalizability of the findings. Future studies could apply the five-category framework developed here to other high-frequency verbs, such as TAKE and HAVE, to build a more comprehensive model of their function in dramatic translation. Second, the analysis is confined to a single translation by Cyril Birch. To distinguish the translator’s personal idiolect from broader translation norms for classical Chinese drama, subsequent research should involve a comparative analysis with other English translations of *Jiaohongji*. Furthermore, this study lacks a comparative analysis against a corpus of native English drama. Incorporating such a reference corpus in future work would provide an essential baseline for more objectively evaluating the stylistic features of the translation, strengthening claims regarding normalization, stylistic balance, and target-audience accessibility. Finally, to enhance methodological rigor, future research should introduce independent coder validation for the functional and qualitative categorizations presented, such as ‘phraseological creativity’ and ‘formulaic status’. This would add a crucial layer of empirical support to the analysis, moving beyond the present researchers’ interpretation.

## Supporting information

S1 Fig Wordcloud of *Mistress and Maid (Jiaohongji).* (JPG)
