# Supplementary figures and images for: A corpus-based analysis on the use of MAKE in sinologist Cyril Birch’s English version of Mistress and Maid (Jiaohongji)
Source: PLoS One. 2026 Jan 8;21(1):e0338015. doi: 10.1371/journal.pone.0338015 (PMC12782422; doi:10.1371/journal.pone.0338015)

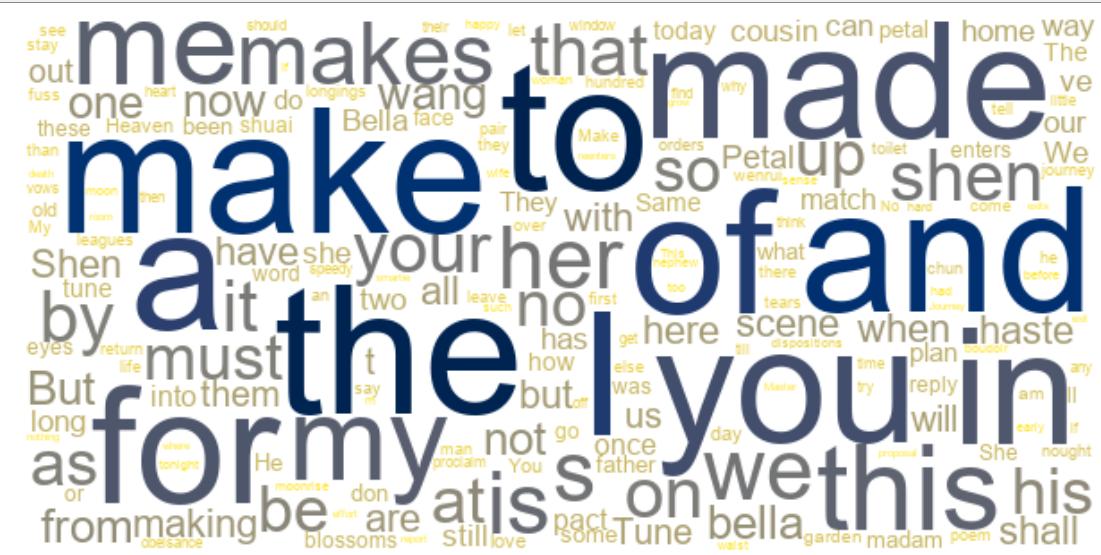

Supplement: S1 Fig — (JPG) [file pone.0338015.s001.jpg]
